# Interpalpebral Fitting Philosophy: A Rarely Used Approach to Keratoconus Management With Corneal Rigid Gas-Permeable (RGP) Contact Lenses

**DOI:** 10.7759/cureus.91961

**Published:** 2025-09-10

**Authors:** Ahmed Almaweri

**Affiliations:** 1 Department of Optometry, Noor Alyemen Eye and ENT Consulting Center, Sana'a, YEM

**Keywords:** case report, corneal lenses, interpalpebral fitting, keratoconus, large palpebral aperture, rigid gas-permeable contact lenses

## Abstract

This unique case report demonstrates an unusual approach to managing keratoconus: the interpalpebral fitting philosophy. This technique is rarely used in contact lens practice. In contrast, the lid-attachment fitting philosophy is more commonly utilized. The interpalpebral fitting is a challenging task concerning lens centration and stability. This report underscores the potential efficacy of the interpalpebral fitting approach in keratoconus patients with specific characteristics, such as large palpebral apertures. A 25-year-old man presented to our center for an eye examination, complaining of blurred vision. Upon examination, both eyes showed signs of keratoconus. The visual acuity without glasses was 20/400 for the right eye and 20/200 for the left eye. Visual acuity with the best correction was as follows: right eye 20/80 with -6.00/-2.50 x 45 and left eye 20/60 with -3.00/-4.00 x 145. Examinations revealed that the patient was suitable for contact lens wear, with no contraindications identified. Corneal rigid gas-permeable contact lenses with an 8.70 mm diameter were fitted according to the interpalpebral fitting strategy. The patient reported good adaptation to the lenses, with acceptable initial comfort. The fitting was excellent, resulting in a marked improvement in visual acuity, achieving 20/20 in each eye. The key takeaway from this study is that the interpalpebral fitting method is a viable option for managing keratoconus in patients with large palpebral apertures.

## Introduction

Keratoconus is a progressive ectatic corneal disorder marked by a thinner, weaker cornea and an abnormally increased curvature, resulting in irregular astigmatism and myopia. Consequently, patients with keratoconus complain of blurry vision, glare, and frequent changes in their eyeglass prescriptions. It develops gradually and may lead to severe vision impairment [1]. Keratoconus exhibits no manifest preference regarding ethnicity or gender. While it is predominantly an ocular condition, it may also be linked to various other ocular and systemic diseases [2].

In cases of mild astigmatism in the initial stages of keratoconus, eyeglasses are helpful. However, as the condition progresses to the advanced stages, glasses serve a very restricted role, and contact lenses take on a major role in improving vision. In this regard, there are several different types of contact lenses that can be used for keratoconus management, and any of these can be used as a starting point based on the severity of the condition and any other related eye considerations [3]. Among these options, rigid gas-permeable (RGP) contact lenses have been the fundamental approach to correcting vision for patients with keratoconus, both before and after gas-permeable materials were developed. They work by creating a layer of tears between the anterior corneal surface and the posterior contact lens surface, which helps to compensate for the irregular shape of the cornea [4]. In fact, corneal RGP lenses have been the most widespread type of contact lens for keratoconus in the past few decades. Over this period, however, the use of scleral lenses has become less common [3]. Even so, there are numerous fitting philosophies and a variety of lens designs to choose from for keratoconus management, and choosing the most suitable technique is a debated topic within the realm of contact lens fitting [5].

The interpalpebral fitting philosophy involves positioning the lens in the space between the open eyelids without it being tucked underneath the eyelids. This technique can be challenging to apply, particularly for patients with keratoconus, as the cone's apex is rarely found at the cornea's geometric center, causing the lens to gravitate towards this apex. Additionally, this type of lens fitting is generally used in specific cases, such as in patients with Graves' disease, where lid retraction occurs, or when there is a filtering bleb formation [6]. In line with these considerations, the interpalpebral fitting philosophy is regarded as a challenging fitting strategy, primarily due to issues linked to lens centration and stability. These difficulties result from the absence of eyelid support for the lens, as well as the relatively smaller lens diameter generally used in this method.

The primary objective of this case study is to document the successful use of the interpalpebral fitting philosophy for managing keratoconus with corneal RGP contact lenses, thereby eliminating the need for lid attachments. By demonstrating this approach, the case report provides significant insights for contact lens practice, especially for professionals who have limited choices and can only use corneal RGP contact lenses for keratoconus management. Additionally, this study contributes a valuable addition to the literature by providing a practical explanation of this fitting philosophy.

## Case presentation

A 25-year-old male presented to our eye center for an examination, complaining of blurred vision. He had been wearing glasses for the past 10 years. His medical history was unremarkable, with no history of vernal keratoconjunctivitis (VKC) and no family history of keratoconus. All eye examinations and interventions were performed at the Ophthalmology and Optometry Department of the Noor Alyemen Eye and ENT Consulting Center in Sana'a, Yemen. These examinations included visual acuity assessment with and without glasses, objective and subjective refraction, slit-lamp examination of the eye and adnexa, fundus examination, and corneal tomography (Pentacam, Oculus, Germany).

Eye examinations revealed keratoconus in both eyes. Upon detailed assessment, the slit-lamp examination exhibited the classic signs of keratoconus in each eye. The fundus findings were unremarkable. The visual acuity without glasses was 20/400 for the right eye and 20/200 for the left eye. Visual acuity with the best correction for the right eye was 20/80 with -6.00/-2.50 x 45, and for the left eye, it was 20/60 with -3.00/-4.00 x 145. Pinhole testing revealed a visual acuity of 20/100 in the right eye and 20/80 in the left eye. Furthermore, a series of measurements was taken. The palpebral fissure height (PFH) was the same in both eyes, measuring 14 mm. The horizontal visible iris diameter (HVID) was 12 mm in both eyes. The vertical visible iris diameter (VVID) was 11 mm in both eyes. The diameter of the pupils was measured using a ruler. In bright lighting, both pupils measured 3 mm, while in dim lighting, they expanded to 5 mm. The tension of the eyelids was normal, neither too tight nor too loose. The tear film was also normal in both quality and quantity. In addition, intraocular pressure (IOP) was measured by a handheld rebound tonometer (ICare Finland Oy, Vantaa, Finland) at 11:00 AM, and the measurements were 8 mmHg for the right eye and 9 mmHg for the left eye.

**Figure 1:**
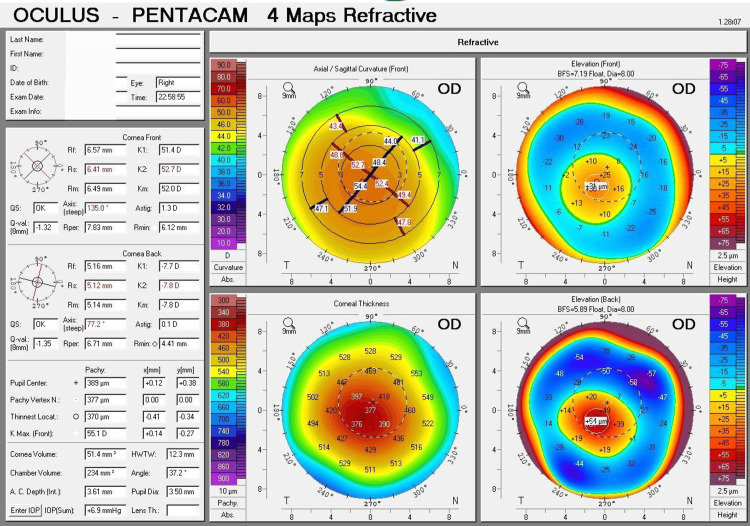
Pentacam corneal tomography of the right eye: refractive four-map analysis Refractive four maps of Pentacam corneal tomography for the right eye demonstrate advanced (Grade 3) keratoconus, as shown by the abnormal ectasia indices. The curvature map displays significant steepening, with a maximum curvature (Kmax) of 55.1 D. The thickness map indicates a markedly thin cornea, with the thinnest point measuring 370 µm. The elevation map shows considerable protrusion, with an anterior elevation of 31 µm.

Tomographic results showed irregularities in the cornea of both eyes. Corneal tomography offers essential information for contact lens fitting decisions, including assessing the severity of the condition and understanding the shape of the keratoconus cone. To further illustrate these findings, the important parameters of the Pentacam four maps were as follows: For the right eye, K1 = 51.4 D (6.57 mm), K2 = 52.7 D (6.41 mm), Km = 52.0 D (6.49 mm), with a maximum curvature (Kmax) of 55.1 D and an astigmatism of 1.3 D. The thinnest location of the cornea was 370 µm; at the center of the pupil, the corneal thickness was 389 µm. The highest point on the front surface of the cornea was elevated by 31 µm, and for the back surface, it was elevated by 64 µm (Figure 1).

**Figure 2:**
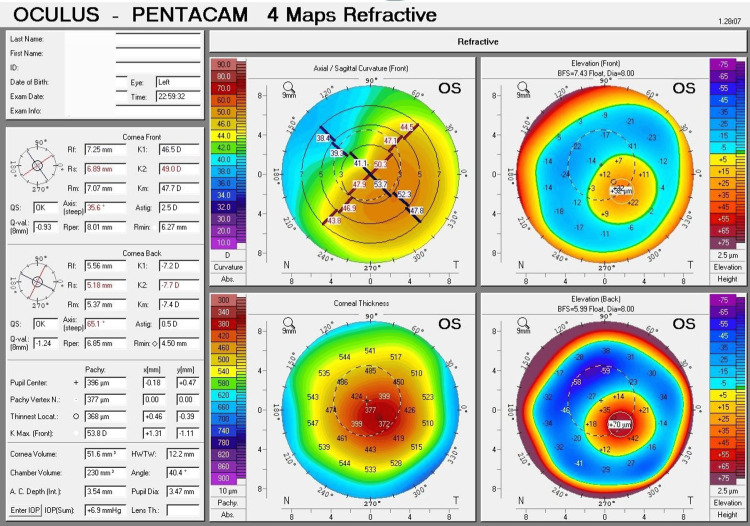
Pentacam corneal tomography of the left eye: refractive four-map analysis. Refractive four maps of Pentacam corneal tomography for the left eye demonstrate moderate (Grade 2) keratoconus, as revealed by the abnormal ectasia values. The curvature map shows marked steepening, with a maximum curvature (Kmax) of 53.8 D. The thickness map presents a notably thin cornea, with the thinnest site measuring 368 µm. The elevation map reveals substantial protrusion, with an anterior elevation of 32 µm.

For the left eye, K1 = 46.5 D (7.25 mm), K2 = 49.0 D (6.89 mm), Km = 47.7 D (7.07 mm), with a maximum curvature (Kmax) of 53.8 D and an astigmatism of 2.5 D. The thinnest location of the cornea was 368 µm; at the center of the pupil, the corneal thickness was 396 µm. The highest point on the front surface of the cornea was elevated by 32 µm, and for the back surface, it was elevated by 70 µm (Figure 2).

After confirming the diagnosis of keratoconus, the patient was prepared to undergo corneal collagen cross-linking (CXL). Following the successful surgical intervention and postoperative follow-up in the cornea specialty clinic, the patient was sent to the contact lens clinic for keratoconus management using rigid gas-permeable contact lenses. After a preliminary examination, the patient was found to be a good candidate for contact lens wear, and there were no contraindications to their use. Subsequently, in the fitting of corneal RGP contact lenses for this patient, a diagnostic trial technique was adopted in accordance with the philosophy of fitting contact lenses without the need for lid attachment (interpalpebral fitting philosophy).

Before the insertion of the first trial lens, topical anesthetic eye drops were used in each eye. This step was undertaken to suppress reflex tearing, thereby enabling a more precise assessment of the contact lens fit. The fitting process was initially attempted with medium- to large-diameter lenses in an effort to achieve lid attachment, but this was unsuccessful due to the patient’s wide palpebral aperture and high upper eyelid position. A smaller lens, with a diameter of 8.70 mm and a base curve equal to the average corneal curvature (K-mean), was then trialed. This choice demonstrated acceptable centration. As a result, the interpalpebral fitting approach was employed as an alternative to the lid-attachment technique.

**Figure 3:**
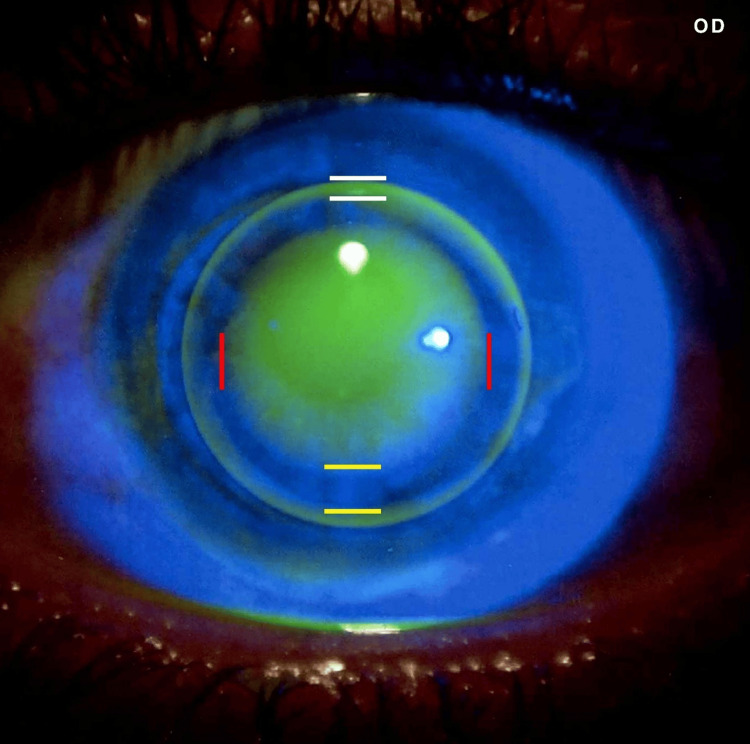
Fluorescein fitting pattern for the right eye. The fluorescein fitting pattern for the right eye demonstrates an optimal fit, identified by proper geometric alignment between the rigid gas-permeable (RGP) lens and the cornea. This pattern adheres to the apical clearance fitting technique; the central and paracentral areas are marked by red lines, the mid-peripheral zone (landing zone) is marked by yellow lines, and the periphery (edge lift) is marked by white lines. This pattern indicates excellent lens centration without lid attachment, with a slightly steep fit demonstrating the interpalpebral fitting philosophy.

**Figure 4:**
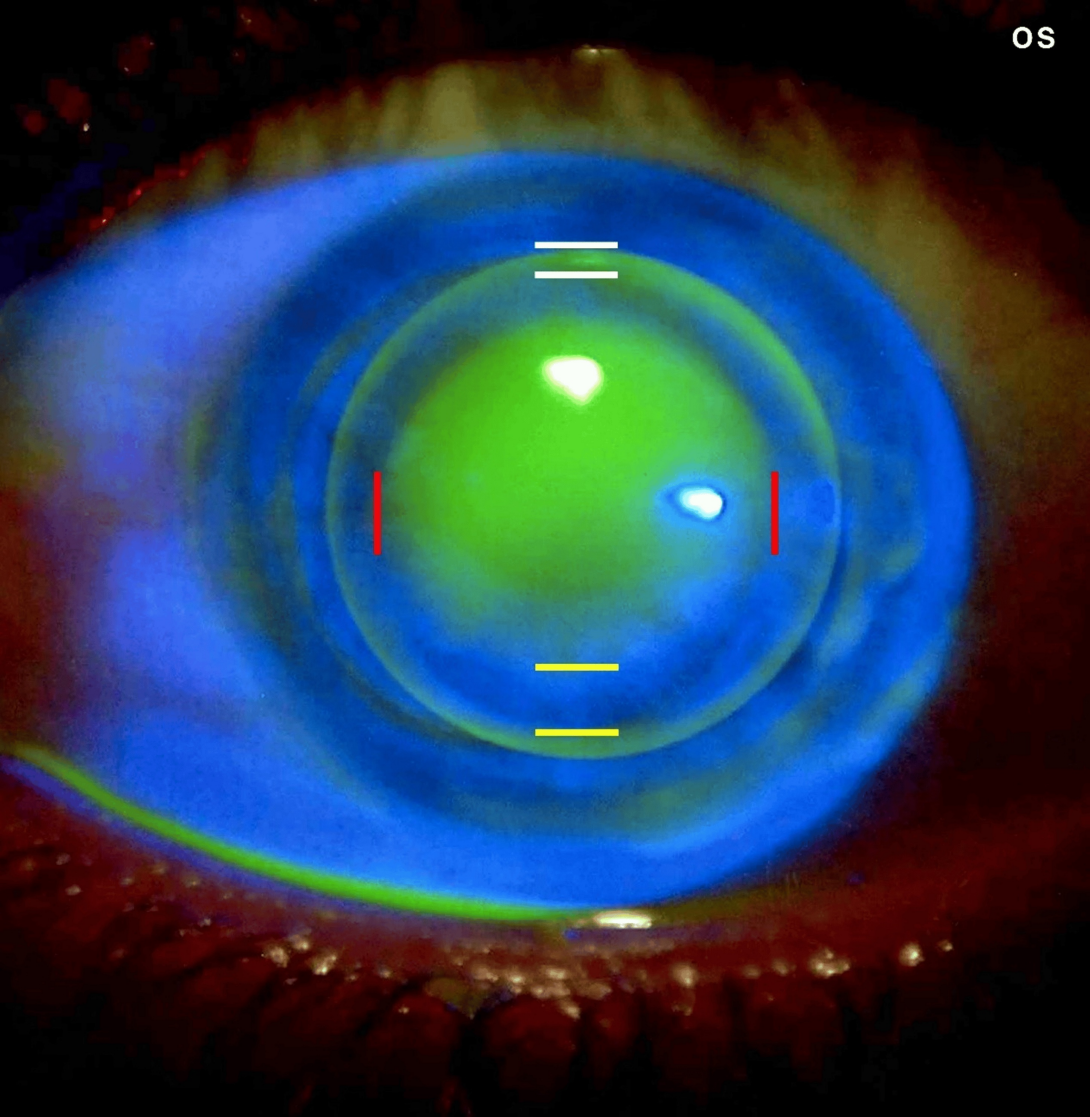
Fluorescein fitting pattern for the left eye. The fluorescein fitting pattern for the left eye demonstrates an optimal fit, characterized by proper geometric alignment between the rigid gas-permeable (RGP) lens and the cornea. This pattern follows the apical clearance fitting method; the central and paracentral areas are marked by red lines, the mid-peripheral zone (landing zone) is marked by yellow lines, and the periphery (edge lift) is marked by white lines. This pattern illustrates that the lens is well-centered, independent of lid attachment, with a slightly steep fit demonstrating an interpalpebral fitting philosophy.

After confirming that the lens was properly centered, the next phase of the fitting assessment focused on lens movement within the eye. This movement was assessed after a blink, and it was determined to be ideal, showing neither restriction nor excessive mobility. Following this, the fitting assessment progressed to checking how well the lens aligned with the cornea. The base curve was initially set equal to the average corneal curvature (K-mean) and then adjusted until an optimal fit was achieved. In this step, the geometric alignment between the contact lens back surface and the anterior corneal surface was evaluated using fluorescein strips with saline solution. Ultimately, the fluorescein pattern showed optimal fitting with apical clearance and a slightly steeper fit, which improved lens stability in the eye and allowed for proper tear flow underneath the lens (Figures 3-4).

To achieve the best visual outcomes, an over-refraction was carefully conducted to determine the accurate lens back vertex power (BVP). To obtain an initial impression of the patient’s comfort and tolerance with the fitted contact lenses, the patient was instructed to wear them for one hour while in the waiting room. After this short-term trial wear, the patient reported a notable decrease in awareness of the lenses, with no manifestations of excessive tearing, pain, or eye irritation.

The final parameters of the corneal RGP contact lenses for the patient were as follows: base curve (6.60 mm), total diameter (8.70 mm), and power (-10.00 D) for the right eye; base curve (6.70 mm), total diameter (8.70 mm), and power (-9.25 D) for the left eye. Visual acuity with RGP lenses was 20/20 in each eye. I requested that the patient's order for contact lenses include a light blue tint for the right lens and a light gray tint for the left lens to facilitate handling and avoid confusion between them.

## Discussion

This case study demonstrates the successful use of corneal RGP contact lenses to manage keratoconus based on the interpalpebral fitting philosophy, particularly in patients with wide palpebral fissures. While it is not a common approach, this method offers a significant advantage for these specific patients. In this clinical scenario, the primary goal of prescribing contact lenses is to improve visual acuity.

The interpalpebral fitting technique is the preferred option for patients with a wide palpebral aperture, high upper eyelid locations, or extremely tight upper eyelids when the attainment of lid attachment is not possible. In such cases, the goal is to enhance corneal adherence while reducing lid interaction. To achieve this, the total diameter of the lens should be smaller, and the fitting should be slightly steeper, but not so excessively steep that tear exchange is restricted [7]. The structure and pattern of the eyelids can affect how well a rigid contact lens is centered and stable on the eye. Additionally, the shape of the eyelids alters the effects of lens design and lens weight. Before fitting the lens, it's important to consider how the lens should interact with the eyelids [8].

The interpalpebral fitting approach was selected for this patient due to the following important considerations: First, the patient had a palpebral fissure height of 14 mm in both eyes, which is considered large. A large palpebral fissure height makes it difficult to achieve lid attachment. Second, the interpalpebral fit, with its focus on apical clearance and lens positioning within the palpebral aperture, helps to avoid excessive pressure on the already compromised cornea. Third, the patient's lifestyle activities did not involve significant physical exertion, such as jumping or high-impact movements, which makes the interpalpebral fitting technique a good choice [5,7].

Interpalpebral lens fitting presents significant benefits. By positioning the lens away from the corneal limbus, it reduces the risk of limbal irritation and minimizes interference with corneal metabolism. This lens placement may also help avoid peripheral corneal staining, particularly at the 3 and 9 o’clock positions [9]. Corneal contact lenses that rub against the eyelid margin exhibit a higher incidence of patient discomfort. Discomfort is the most common complaint among corneal RGP contact lens users, occurring as a consequence of the interaction between the eyelid margin and the lens edge. In this case, the interpalpebral fitting technique was used to help minimize discomfort and improve lens tolerance [10,11].

Three primary techniques exist for distributing contact lens weight on the cornea: apical touch, apical clearance, and three-point touch. In this patient's case, the apical clearance fitting method was applied. With apical clearance, the corneal RGP lens primarily rests on the mid-peripheral cornea (landing zone) and vaults over the central and paracentral cornea. This fitting typically involves a slightly steeper lens design [12,13]. A slightly steep fitting relationship facilitates the positioning of the lens within the interpalpebral space [14]. In this approach, the fitting is made slightly steep, just enough to allow for tear exchange. This is a key part of the interpalpebral fitting technique. This pattern enhances lens stability on the cornea by reducing movement; in addition, the suction force of the lens further reinforces this stability. This study draws attention to the fundamental role of the suction forces created between the cornea, tears, and contact lenses in maintaining lens stability. It indicates that a deeper understanding of these forces can contribute to innovative designs that improve the stability of corneal contact lenses [15,16].

In contact lens practice, the interpalpebral fitting philosophy is an uncommon approach. Typically, the lid attachment technique is used for the trial and fitting of patients selected for corneal RGP contact lenses. If the fit is unsatisfactory or unconvincing, the lens is switched to a scleral RGP lens.

The lid attachment fitting philosophy is an alternative method to the interpalpebral fitting approach, and it is the most commonly used strategy for fitting corneal RGP lenses for keratoconus patients. The lid attachment procedure allows the upper eyelid to attach to the lens edge, which helps with lens stability. The advantages of this fitting include better centration, stability, good vision, and increased comfort due to the lens edge being located beneath the eyelid. However, it is important to take into account the geometry of the eyelids. This method usually utilizes a larger diameter with a flatter base curve. This approach is often recommended for patients with normally or low-positioned upper eyelids and those who have a physically active daily life [4,5,8].

Pentacam corneal tomography data were used to optimize the contact lens fit for this patient. Corneal tomography is an important resource in fitting RGP contact lenses, as it offers comprehensive details about the cornea. Tomography captures the entire cornea, highlighting irregularities and asymmetries that keratometry may overlook. This is especially important for fitting challenging corneas, such as those affected by keratoconus or those that have undergone refractive surgery [17,18].

The patient was advised to participate in regular follow-up appointments. Subsequent to the fitting procedure, the patient was scheduled to return for thorough evaluations. Once the fitting was confirmed to meet satisfactory standards, follow-up visits were arranged at six-month intervals. Throughout all previous visits, there were no reported complications or adverse events. During each appointment, assessments were conducted of the patient’s vision, lens fitting quality, ocular health, and adherence to the recommended regimen. In this case study, the patient reported significant improvement in performing daily tasks and work-related activities after the fitting of corneal RGP contact lenses. These improvements, supported by his feedback during follow-up assessments, emphasize the positive impact of this management approach on visual function and overall quality of life.

This case study has some limitations. First, it focuses on just one case, which makes it difficult to apply the findings more broadly. Second, the absence of a comparative analysis makes it difficult to highlight the specific advantages of the interpalpebral fitting philosophy over the lid-attachment technique. Third, the lack of patient-reported outcome measures (PROMs) hinders a full exploration of patients' individual experiences. Moreover, relying on just one follow-up appointment fails to offer thorough insight into the course of the condition and any potential issues that might arise later on.

This case study suggests that the interpalpebral fitting philosophy is a successful management strategy for keratoconus patients with wide palpebral apertures. Future studies should investigate this approach with a larger number of cases. Additionally, it is important to conduct comparative studies on interpalpebral and lid-attachment fitting techniques to identify the best approach for various keratoconus patient characteristics. Future studies should evaluate enhancements in the quality of life through the utilization of established PROMs, such as the National Eye Institute Visual Function Questionnaire-25 (NEI VFQ-25) or the Ocular Surface Disease Index (OSDI). Moreover, a follow-up duration of no less than 12 to 24 months is essential to confirm treatment results and identify any delayed undesirable effects.

## Conclusions

This case report demonstrates the successful application of the interpalpebral fitting philosophy, which is less commonly used compared to conventional lid attachment techniques. The interpalpebral fitting, characterized by a slightly steeper fit, ensures optimal lens stability and centration within the interpalpebral space, minimizing lid interaction and enhancing patient comfort. As a result of the patient's wide palpebral aperture, achieving lid attachment with the corneal RGP lens is challenging. This study emphasizes the potential benefits of the interpalpebral fitting strategy for managing keratoconus in specific patients, particularly those with wide palpebral aperture fissures.
